# Polyelectrolyte Microcapsule-Assembled Colloidosomes: A Novel Strategy for the Encapsulation of Hydrophobic Substances

**DOI:** 10.3390/polym17141975

**Published:** 2025-07-18

**Authors:** Egor V. Musin, Alexey V. Dubrovskii, Yuri S. Chebykin, Aleksandr L. Kim, Sergey A. Tikhonenko

**Affiliations:** 1 Moscow Polytechnic University (Moscow Polytech), Bolshaya Semyonovskaya St., 38, Moscow 107023, Russia; eglork@gmail.com; 2 Institute of Theoretical and Experimental Biophysics Russian Academy of Science, Institutskaya St., 3, Puschino 142290, Moscow Region, Russia; dav198@mail.ru (A.V.D.); kobepoftruth@gmail.com (Y.S.C.)

**Keywords:** colloidosomes, polyelectrolyte microcapsules, hydrophobic substances, encapsulation

## Abstract

The encapsulation of hydrophobic substances remains a significant challenge due to limitations such as low loading efficiency, leakage, and poor distribution within microcapsules. This study introduces a novel strategy utilizing colloidosomes assembled from polyelectrolyte microcapsules (PMCs). PMCs were fabricated via layer-by-layer (LbL) assembly on manganese carbonate (MnCO_3_) or calcium carbonate (CaCO_3_) cores, followed by core dissolution. A solvent gradient replacement method was employed to substitute the internal aqueous phase of PMCs with kerosene, enabling the formation of colloidosomes through self-assembly upon resuspension in water. Comparative analysis revealed that MnCO_3_-based PMCs with smaller diameters (2.5–3 µm vs. 4.5–5.5 µm for CaCO_3_) exhibited 3.5-fold greater stability, attributed to enhanced inter-capsule interactions via electrostatic and hydrophobic forces. Confocal microscopy confirmed the structural integrity of colloidosomes, featuring a liquid kerosene core encapsulated within a PMC shell. Temporal stability studies indicated structural degradation within 30 min, though 5% of colloidosomes retained integrity post-water evaporation. PMC-based colloidosomes exhibit significant application potential due to their integration of colloidosome functionality with PMC-derived structural features—semi-permeability, tunable shell thickness/composition, and stimuli-responsive behavior—enabling their adaptability to diverse technological and biomedical contexts. This innovation holds promise for applications in drug delivery, agrochemicals, and environmental technologies, where controlled release and stability are critical. The findings highlight the role of core material selection and solvent engineering in optimizing colloidosome performance, paving the way for advanced encapsulation systems.

## 1. Introduction

Encapsulation represents a pivotal strategy in modern science for enhancing the functional properties of diverse substances, owing to the high adaptability of encapsulation systems to specific applications [1,2]. By enclosing active compounds within a protective shell, encapsulation improves their stability, shields them from environmental factors, and enables their controlled release over time [1,2,3]. Furthermore, current encapsulation technologies enhance the solubility, bioavailability, and compatibility of compounds with various matrices, while isolating reactive or hazardous substances to ensure operational safety [4,5,6]. These advancements have driven the widespread adoption of encapsulation across multiple industries: in medicine (e.g., drug delivery systems, cancer therapy, and regenerative medicine) [2,7,8,9]; the food industry (improving nutrient stability, such as vitamins and probiotics; controlling flavor release; and enhancing sensory qualities) [1,10]; agriculture (delivering pesticides, fertilizers, and plant growth regulators) [11]; and environmental science (water treatment and controlled release of cleaning agents) [12,13].

Encapsulation methods utilize diverse matrices, broadly categorized as organic or inorganic, to protect and deliver active substances [14,15]. Among these encapsulation methods, polyelectrolyte microcapsules (PMCs) fabricated via layer-by-layer (LbL) technology warrant particular attention. These microcapsules feature a multilayered structure formed by the alternating deposition of oppositely charged polyelectrolytes onto micron-sized core particles. Following core dissolution, hollow microcapsules are obtained, which can encapsulate a wide range of substances, including proteins, enzymes, hormones, and nanoparticles [16,17,18,19]. However, encapsulating water-insoluble (or poorly water-soluble) substances poses a unique challenge, as hydrophobic molecules are immiscible with water, precluding the use of standard protocols reliant on water-soluble polyelectrolytes [20,21].

One straightforward approach for encapsulating hydrophobic compounds into PMCs involves replacing water with an organic solvent, enabling the hydrophobic substance to permeate the capsule shell. For instance, Changchun Deng et al. [22] proposed a method where the ethanol concentration in the incubation solution was gradually increased. Upon reaching maximum ethanol concentration, toluene was incrementally introduced. The hydrophobic compound was then added to the incubation solution, allowed to permeate the PMC shell, and the loaded capsules transferred into an aqueous medium. While this method is effective for encapsulating low-molecular-weight compounds, high-molecular-weight substances cannot penetrate the PMC shell, hindering their encapsulation.

Radtchenko et al. addressed the encapsulation of poorly water-soluble (PWS) compounds by employing PMCs with a polarity gradient [23]. These capsules were prepared using a core containing polystyrene sulfonate. After core dissolution, the residual polyelectrolyte within the capsule retained abundant free ionic groups, establishing a polar gradient between the capsule’s internal volume and the external medium. To encapsulate PWS compounds, the authors dissolved the substance in acetone, incubated it with the capsule suspension, and allowed the acetone to evaporate. The polarity gradient drove the precipitation of PWS compounds inside the capsules. However, this method shares the limitation observed in Changchun Deng et al.’s work. Additionally, it exhibits a low loading efficiency, as a significant portion of the compound remains outside the capsules post-acetone evaporation.

Rongcong Luo et al. proposed an approach that partially addressed the aforementioned challenges [24]. In this method, the authors utilized spherical calcium carbonate (CaCO_3_) microparticles as cores, which were coated with a layer of poly(lactic-co-glycolic acid) (PLGA) dissolved in acetone or N-methyl-2-pyrrolidone and which contained a hydrophobic compound (ibuprofen). Subsequently, layers of poly(allylamine hydrochloride) (PAH) and poly(styrene sulfonate) (PSS) polyelectrolytes were deposited onto the PLGA-coated CaCO_3_ microparticles. The key distinction of this method from previous approaches lies in its ability to incorporate the target substance into the PMCs during shell formation. Increasing the number of shell layers prolongs the release time of the encapsulated substance. However, this method cannot be universally applied, as the encapsulation of compounds within polyelectrolyte layers may compromise their functional activity.

The aforementioned approaches face limitations such as a low loading efficiency, potential leakage/evaporation during preparation, and difficulties in achieving uniform distribution within the capsules. Addressing these shortcomings remains critical for expanding the application of polyelectrolyte microcapsules in hydrophobic substance encapsulation. Consequently, the objective of this work is to develop a novel method for encapsulating hydrophobic compounds using polyelectrolyte microcapsules templated on inorganic cores of manganese carbonate and calcium carbonate.

## 2. Materials and Methods

Polystyrene sulfonate sodium (PSS) and polyallylamine hydrochloride (PAH) with a molecular mass of 70 kDa Sigma (Merck KGaA, Darmstadt, Germany) and with a purity of ≥99% were obtained. Rhodamine B and fluorescein isothiocyanate (FITC) were also obtained from Sigma (Merck KGaA, Darmstadt, Germany) with a purity of ≥98%; ethylenediaminetetraacetic acid (EDTA), calcium chloride (CaCl_2_ × 2H_2_O), manganese chloride, sodium chloride, kerosene, isopropanol, sodium sulfate, and sodium carbonate were supplied by Reahim (Reahim AO, St. Petersburg, Russia) with a minimum purity of 99.9%.

### 2.1. Preparation of Fluorescently Labeled PAH

FITC was slowly added to a solution of polyelectrolyte (10 mg/mL) in 50 mM borate buffer, pH 9.0, while stirring the mixture at 300–400 rpm. The FITC and PAH were fused in a molar ratio of 1:100. After that, the solution (15 mL) was incubated for 1.5–2 h. Once the incubation period was over, we dialyzed the solution against water (10 L) overnight, ensuring that there was enough time for the labeled PAH to be purified and ready for use in further experiments.

### 2.2. Preparation of CaCO_3_ and MnCO_3_ Microspherulites

**Figure 1:**
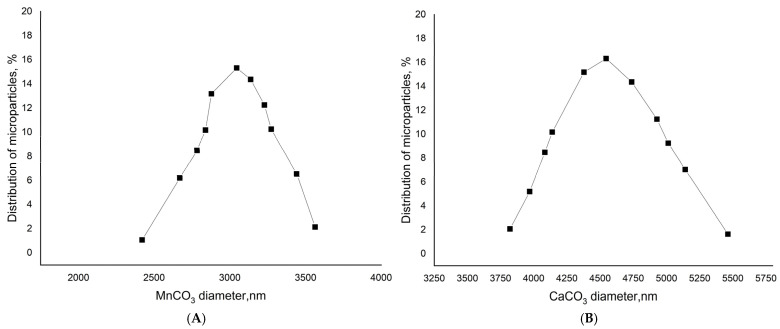
Size distribution of MnCO_3_ (**A**) and CaCO_3_ (**B**) particles.

While stirring the 0.33 M Na_2_CO_3_, the 0.33 M CaCl_2_ (or MnCl_2_) was added [25]. The stirring time was 30 s. The suspension was maintained until the complete precipitation of the formed particles. The process of the “ripening” of the microspherolites was controlled with the help of a light microscope. Their shape and the presence of aggregates were assessed at 1000× magnification using immersion oil; spherulites exhibiting a regular spherical shape and no aggregation were considered successfully formed. Then, the supernatant was decanted and the precipitate was washed with water and used to prepare PMCs. The 10 million microparticles were obtained with an average diameter of 4.5 ± 1 μm in the case of CaCO_3_ and 3 ± 1 μm in the case of MnCO_3_ (Figure 1).

### 2.3. Preparation of Polyelectrolyte Microcapsules Formed on CaCO_3_ and MnCO_3_

The polyelectrolyte microcapsules were obtained by layer-by-layer adsorption of the negatively or positively charged polyelectrolytes onto CaCO_3_ (or MnCO_3_) microspherulites, followed by the dissolution of CaCO_3_. At the moment of the dissolution of the CaCO_3_ core, the inner space of the PMC was filled by the interpolyelectrolyte complex [26]. Layer-by-layer adsorption of PAH and PSS on the CaCO_3_ microspherulite surface was carried out in the polyelectrolyte solutions (concentration of 2 mg/mL + 0.5 M NaCl). After each adsorption (15 min), the CaCO_3_ particles with adsorbed polyelectrolytes were triple-washed with a 0.5 M NaCl solution, which was necessary to remove unabsorbed polymer molecules. The particles were separated from the supernatant by centrifugation. After applying the required number of layers, the carbonate cores were dissolved in a 0.2 M EDTA solution for 12 h. The resulting capsules were washed three times with water to remove core decay products. The final shell composition was (PAH/PSS)_3_, where PAH was the first shell layer (closer to the core) and PSS was the outer shell layer. The microcapsules (on CaCO_3_) were obtained with an average diameter of 4.5 ± 1 μm. The microcapsules (on MnCO_3_) were obtained with an average diameter of 3 ± 1 μm. The size and number of microcapsules were measured using the dynamic light scattering method on a Zetasizer nano ZS device (Malvern, UK).

### 2.4. Fabrication of Colloidosomes Based on Polyelectrolyte Microcapsules

Ten million polyelectrolyte microcapsules (PMCs) were incubated for 15 min in a water/isopropanol (1:10 *v/v*) solution. The PMC samples were then centrifuged, and the supernatant was removed. This procedure was repeated while incrementally increasing the isopropanol concentration to ratios of 2:10, 3:10, 4:10, etc., until the sample was fully incubated in pure isopropanol. This stepwise solvent replacement was essential to eliminate water from the PMCs. Subsequently, the microcapsules were subjected to an analogous protocol using a kerosene solution. The kerosene/isopropanol ratio was progressively increased (1:10, 2:10, etc.) in 15 min incubation steps, ultimately yielding PMCs dispersed in pure kerosene.

Following the replacement of the aqueous medium with kerosene, the PMCs were precipitated via centrifugation, and the supernatant was discarded. A small volume of kerosene-containing PMC sediment was mixed with water and resuspended using a vortex mixer for 10 min. This process resulted in the formation of colloidosomes based on polyelectrolyte microcapsules.

### 2.5. Microscopy

Polyelectrolyte microcapsules were placed between two coverslips in a drop of water medium at a concentration of 2 × 10^6^ particles/mL. Images were acquired with the Leica TCS SP5 confocal system (Leica Microsystems, Wetzlar, Germany) as a single image or a Z-stack using HCX PL APO lambda blue 63.0 × 1.40 OIL UV. Image resolution—512 × 512 px, optical resolution—70 nm/px, and spatial resolution—140 nm. Rhodamine B fluorescence was excited using a 550 nm line of the argon laser (the intensity was set to 4–10% of the maximum). The fluorescence emission was collected at the range of 570–620 nm. The thickness of the optical section in the Z-stack was 0.4–0.9 µM. Images were acquired at a 400 Hz scan speed using 3× line averaging to reduce noise. Image processing was performed using FiJi (ImageJ 1.53t) software.

The study of the samples’ “bright-field microscopy” was carried out in a Zeiss Axiovert 200M Cell Observer microscope (Zeiss, Moscow, Russia). To obtain fluorescent micrographs, excitation with light with a wavelength in the region of 480–490 nm was used, using a combination of light filters.

## 3. Results and Discussion

**Figure 2:**
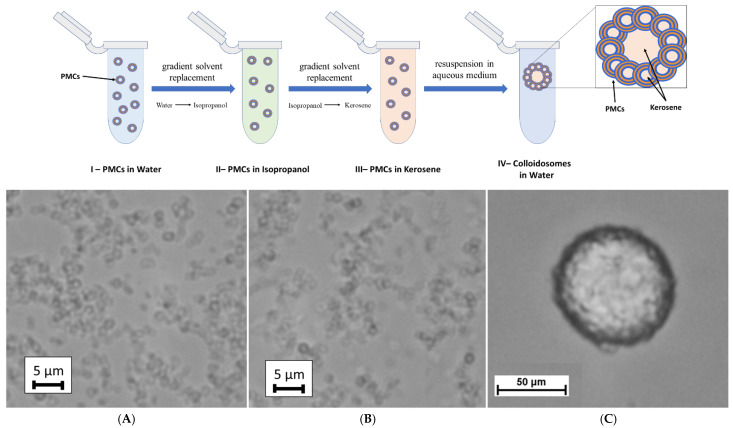
The steps of the colloidosome fabrication method and micrographs of polyelectrolyte microcapsules (PMCs) in different incubation media after gradient replacement of the internal liquid phase. (**A**) PMCs in water prior to gradient replacement; (**B**) PMCs in kerosene post-gradient replacement; (**C**) colloidosomes based on polyelectrolyte microcapsules upon resuspension in water.

To encapsulate hydrophobic substances using polyelectrolyte microcapsules (PMCs), a colloidosome-based approach was proposed [27]. Previously, Li et al. [28] demonstrated the feasibility of fabricating PMCs via Pickering emulsions—disperse systems (e.g., oil-in-water) stabilized by solid particles adsorbed at the phase interface without forming a closed shell. The authors employed surface-treated colloidal silica (15 ± 2 nm) with a positive surface charge, modified using poly(styrene sulfonate) (PSS), to generate oil-in-water Pickering emulsions. Sequential adsorption of polyelectrolytes onto the resulting xylene-in-water emulsions yielded PMCs with diameters of 50–100 µm, encapsulating xylene within an aqueous shell. In this work, we adopted this approach but integrated it with the method proposed by Changchun Deng et al. [22], wherein the internal aqueous phase of the PMCs was replaced with an organic solvent. Thus, polyelectrolyte microcapsules (PSS-based) were formed. The steps of our colloidosome fabrication method based on PMCs are illustrated in Figure 2.

The first stage of the colloidosome fabrication method involves replacing the aqueous phase inside the PMCs with kerosene via gradient solvent replacement. To achieve this, the isopropanol concentration in the incubation solution was incrementally increased to its maximum value, followed by the gradual introduction of kerosene at rising ratios (1:10, 2:10, etc.). This resulted in discrete PMCs dispersed in kerosene, with the organic solvent retained as the internal phase (Figure 2B). Subsequently, the microcapsules were centrifuged to form a pellet, the supernatant was discarded, and the sediment was resuspended in an aqueous medium. Vigorous vortex agitation for 10 min promoted the self-assembly of PMCs into spherical colloidosomes (Figure 2C), encapsulating kerosene droplets. The stabilization of the colloidosomes was mediated by the polyelectrolyte shell of the PMCs, which facilitated inter-capsule interactions: electrostatic forces and hydrophobic contacts collectively established a stable three-dimensional architecture.

**Figure 3:**
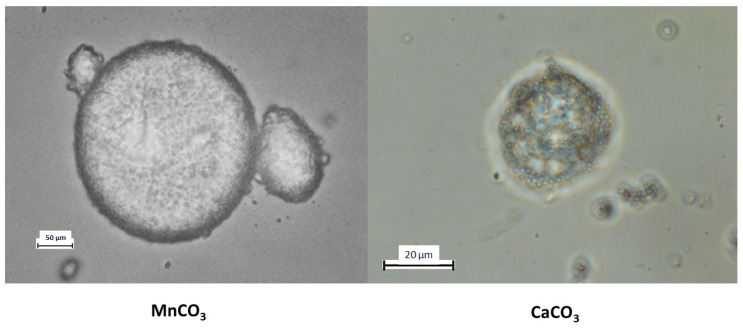
Micrographs of colloidosomes based on PMCs templated on MnCO_3_ particles and PMCs templated on CaCO_3_ particles.

In this study, two types of PMCs were employed for colloidosome fabrication: those templated on MnCO_3_ particles and those templated on CaCO_3_ particles. The results are presented in Figure 3.

**Figure 4:**
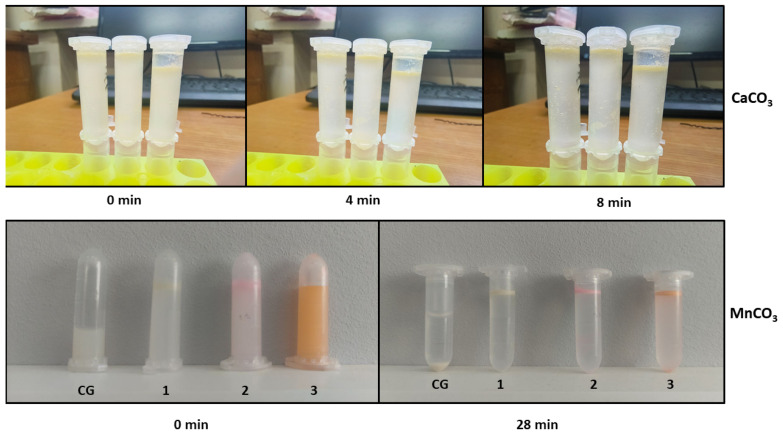
Stability of colloidosomes based on PMCs templated on MnCO_3_ particles and PMCs templated on CaCO_3_ particles. 1—Unlabeled PMCs; 2—Rhodamine B-labeled PMCs; 3—FITC-labeled PMCs.

As shown in the figure, colloidosomes derived from MnCO_3_-templated PMCs exhibit a regular structure with a distinct shell composed of densely packed PMCs. In contrast, colloidosomes formed using CaCO_3_-templated PMCs display a disordered morphology, low packing density, and structural instability. These observations are corroborated by the stability duration of kerosene-in-water emulsions prepared with these PMCs, as illustrated in Figure 4.

Figure 4 demonstrates that colloidosomes derived from MnCO_3_-templated PMCs exhibit 3.5-fold greater stability compared to those based on CaCO_3_-templated PMCs. This disparity is attributed to morphological differences between the systems: MnCO_3_-templated PMCs have smaller diameters (2.5–3 µm) compared to CaCO_3_-templated PMCs (4.5–5.5 µm). The reduced diameter increases the specific surface area, enhancing inter-capsule interactions. This effect amplifies electrostatic and hydrophobic forces at the phase interface, thereby improving structural stability. The lower stability of CaCO_3_-based colloidosomes may arise from PMC agglomeration during the replacement of the internal aqueous phase with kerosene (Supplementary Materials, Figure S1). Given the superior stability of MnCO_3_-based colloidosomes, further investigations should prioritize these structures.

**Figure 5:**
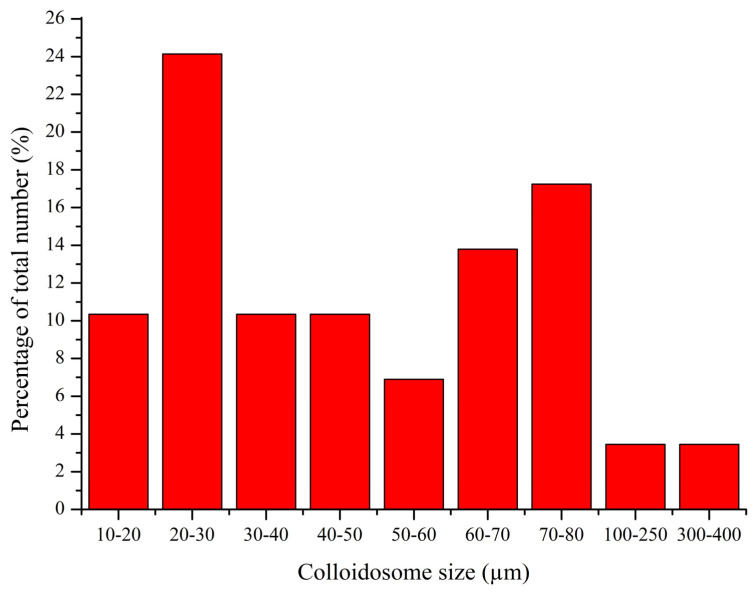
Size distribution of colloidosomes based on PMCs templated on MnCO_3_ particles.

Colloidosomes fabricated using manganese carbonate (MnCO_3_)-templated polyelectrolyte microcapsules exhibit a broad size range (13–450 µm). The polydispersity of the system, including the particle diameter distribution, is presented in Figure 5.

The results presented in Figure 5 confirm the morphological heterogeneity of the studied structures. The observed size variation may arise from variability in the synthesis conditions, such as the particle aggregation rate and local differences in the surface tension at the kerosene/water interface.

**Figure 6:**
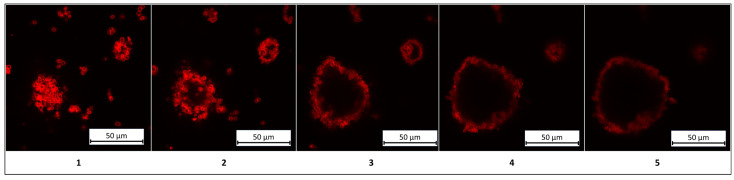
Confocal images of colloidosomes based on polyelectrolyte microcapsules (PMCs). Images (**1**–**5**) correspond to sequential optical sections (Z-stacks) of the sample, acquired at varying depths to visualize the three-dimensional organization of the structures.

A critical aspect of this study involves verifying the structural identity of the systems as colloidosomes, defined by the presence of a liquid core (kerosene) and the absence of polyelectrolyte microcapsules within the core. To resolve this, confocal microscopy was employed to visualize the spatial distribution of PMCs. The results are detailed in Figure 6.

**Figure 7:**
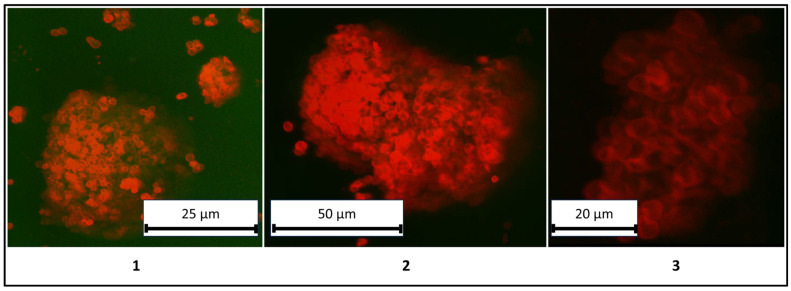
Three-dimensional rendered confocal images of colloidosomes based on polyelectrolyte microcapsules (PMCs). (**1**–**3**): Distinct samples synthesized under identical conditions.

The results presented in Figure 6 demonstrate a clear differentiation between the liquid core (kerosene) and the shell composed of PMCs, confirming the classification of these structures as colloidosomes. For enhanced clarity, three-dimensional rendered images of the colloidosomes were reconstructed and are shown in Figure 7.

Nevertheless, these structures could alternatively be classified as Pickering emulsions. A Pickering emulsion is an emulsion stabilized by solid particles rather than conventional surfactants [29]. Unlike colloidosomes, Pickering emulsions lack additional stabilization via covalent or electrostatic bonds, resulting in metastable assemblies prone to coalescence over time. A similar behavior is observed in the particles described in this work. However, the structures reported here consist of 3-micron polyelectrolyte microcapsules (PMCs) possessing both positive and negative charges, as well as the capacity to form hydrophobic interactions. Therefore, we propose classifying these structures as colloidosomes, as their stabilization is mediated by synergistic electrostatic and hydrophobic forces.

**Figure 8:**
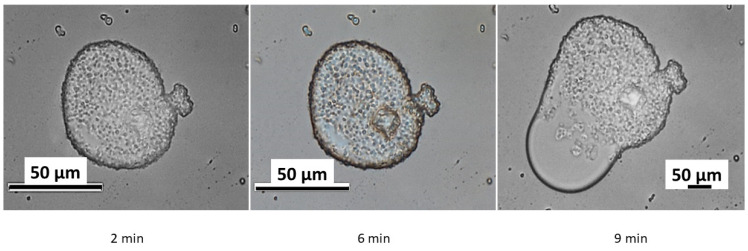

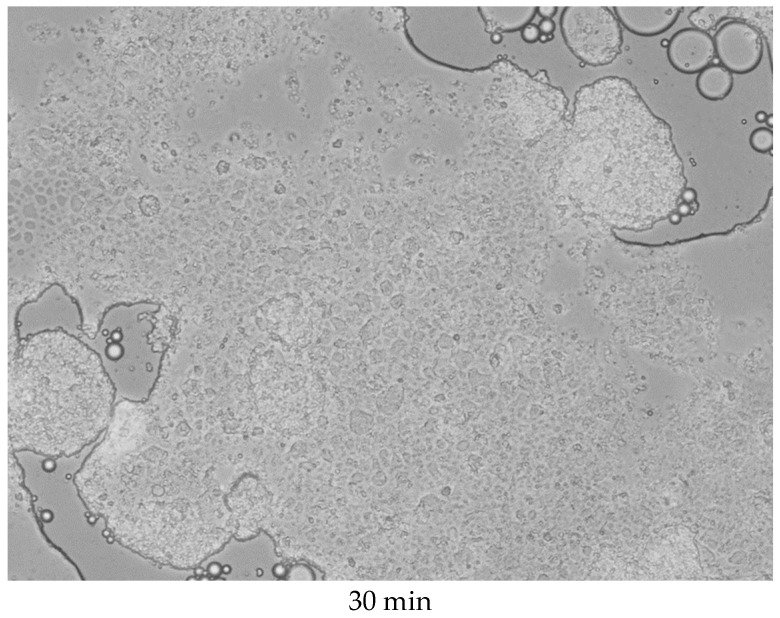
Degradation of colloidosomes over time.

As previously established, the colloidosomes exhibit stability for approximately 30 min. To assess their structural integrity over time, long-term microscopic monitoring was conducted. The results are presented in Figure 8.

As shown in the figure, localized breaches in the shell integrity are observed after 6 min of incubation, leading to the partial release of kerosene from the internal cavity. By 9 min, intensified kerosene release coincides with a loss of structural integrity in the colloidosomes. After 30 min, complete disintegration occurs, resulting in the coalescence of kerosene droplets into a macroscopic phase separated from water. During this process, the capsules either distribute uniformly within the kerosene layer or concentrate at the phase interface, likely due to surface tension effects. Nevertheless, re-shaking the solution induces the re-self-assembly of the colloidosomes.

**Figure 9:**
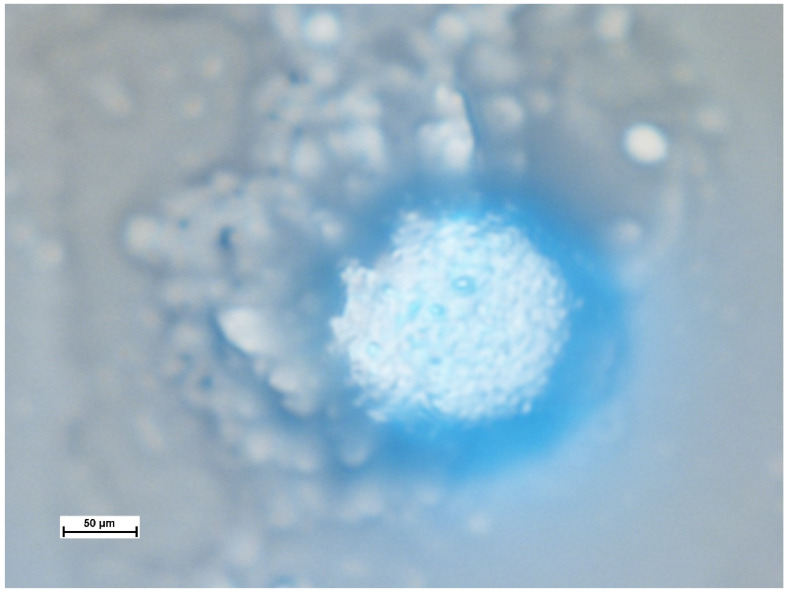
Fluorescence image of a colloidosome after water evaporation from the sample.

However, not all samples exhibited time-dependent degradation. In fewer than 5% of cases, structural integrity was preserved even after water evaporation from the sample (Figure 9).

As shown in the image, the three-dimensional structure of the colloidosome remains intact, with fluorescence emission observed in the 310–420 nm range. This emission corresponds to the fluorescence peak of kerosene [30], confirming that kerosene is retained within the colloidosome.

Based on the results described above, it can be assumed that this technology may be promising, since the capsules within the colloidosome are expected to retain their properties: semi-permeability, permeability to low-molecular-weight substances, and impermeability to high-molecular-weight substances; the immobilization of molecules inside the capsules, including metal particles, semiconductors, etc.; and the gradual release of the encapsulated substance into the medium. Due to these properties, in the future, such colloidosomes could be applied as a multifunctional material for various tasks, for example, encapsulating metal particles in PMCs that can be controlled by a magnetic field to destabilize colloidosomes or to create localized heating of the colloidosome shell, thereby generating a temperature gradient inside the colloidosome relative to the aqueous phase.

## 4. Conclusions

This study developed a method for fabricating colloidosomes based on polyelectrolyte microcapsules (PMCs) using manganese carbonate (MnCO_3_) and calcium carbonate (CaCO_3_) particles as templates. The replacement of the internal aqueous phase of PMCs with kerosene via solvent gradient replacement enabled the formation of stable dispersions, which self-assembled into spherical structures upon resuspension in water. Colloidosome stabilization is hypothesized to arise from synergistic electrostatic and hydrophobic interactions between PMCs. MnCO_3_-based colloidosomes exhibited 3.5-fold greater stability compared to CaCO_3_-based counterparts, attributed to their smaller particle size (2.5–3 µm vs. 4.5–5.5 µm) and, consequently, higher specific surface area, which amplified inter-capsule interactions. Confocal microscopy verified the structural identity of colloidosomes, confirming the presence of a liquid core (kerosene) encapsulated within a polyelectrolyte shell. Temporal degradation studies revealed initial shell breaches at 6 min, progressing to complete kerosene droplet coalescence into a macroscopic phase within 30 min. However, 5% of colloidosomes retained structural integrity even after water evaporation.

The developed colloidosome technology offers significant potential for applications requiring hydrophobic substance encapsulation. This approach overcomes the limitations of existing methods, such as low loading efficiency and uneven payload distribution. It is particularly promising for encapsulating high-molecular-weight hydrophobic compounds, which cannot permeate traditional PMC shells. The colloidosomes are fabricated based on polyelectrolyte microcapsules, retaining the unique properties inherent to PMCs: a semi-permeable PMC shell; the controlled thickness and composition of the PMC shell, enabling its customization for specific applications; and the capacity of the shell to respond to external stimuli under specific conditions. These structural and functional attributes ensure the adaptability of the PMC-based colloidosomes to targeted tasks, leveraging the tunable physicochemical characteristics of the polyelectrolyte multilayer assembly. Due to their ability to form stable self-assembled architectures, these colloidosomes could advance applications in drug delivery, agrochemicals, and environmental technologies, where controlled release and structural durability are paramount. Further development requires strategies to prolong structural integrity, explore controlled release profiles in response to environmental cues, and validate encapsulation efficiency for specific high-value hydrophobic payloads.

## Data Availability

Data is contained within the article.

## Supplementary Materials

The following supporting information can be downloaded at: https://www.mdpi.com/article/10.3390/polym17141975/s1, Figure S1: Incubation of PMCs, formed on CaCO_3_ particles, in kerosene solution.
