# Supplementary figures and images for: Polyelectrolyte Microcapsule-Assembled Colloidosomes: A Novel Strategy for the Encapsulation of Hydrophobic Substances
Source: Polymers (Basel). 2025 Jul 18;17(14):1975. doi: 10.3390/polym17141975 (PMC12300669; doi:10.3390/polym17141975)

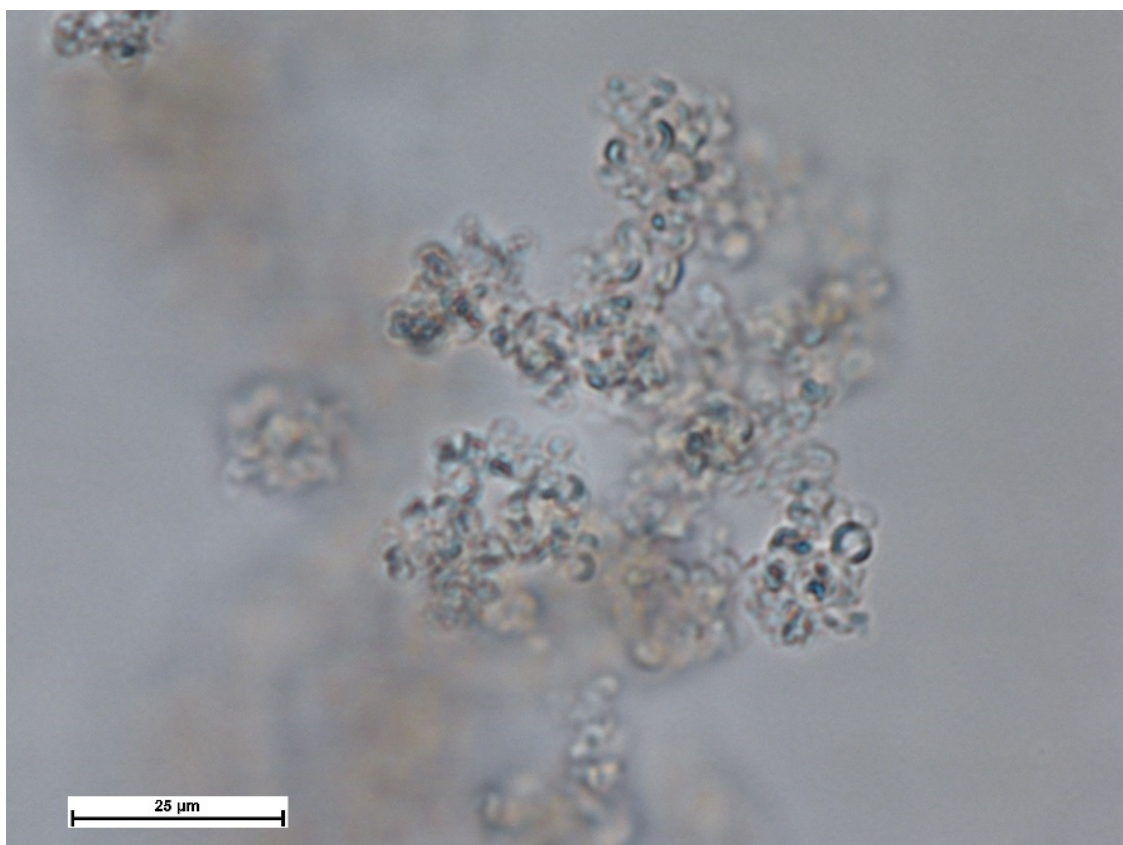

Figure S1. Incubation of PMCs, formed on  $\text{CaCO}_3$  particles, in kerosene solution.

Supplement: Supplementary file 1 [file polymers-17-01975-s001.zip › polymers-3765676-supplementary.pdf]
